# The association of pericardial fat and peri-aortic fat with severity of nonalcoholic fatty liver disease

**DOI:** 10.1038/s41598-022-18499-9

**Published:** 2022-08-18

**Authors:** Chun-Wei Lee, Chun-Ho Yun, Wen-Hung Huang, Ta-Chuan Hung, Cheng-Ting Tsai, Jen-Yuan Kuo, Cheng-Huang Su, Han-En Cheng, Chung-Lieh Hung, Charles Jia-Yin Hou

**Affiliations:** 1 Cardiovascular Division, Department of Internal Medicine, MacKay Memorial Hospital, MacKay Medical College, New Taipei City, Taiwan, ROC; 2 MacKay Junior College of Medicine, Nursing and Management, Taipei, Taiwan, ROC; 3 Department of Medicine, Mackay Medical College, New Taipei City, Taiwan, ROC; 4 Institute of Public Health, National Yang Ming Chiao Tung University, Taipei, 112304 Taiwan, ROC; 5 Institute of Biomedical Sciences, Mackay Medical College, New Taipei City, Taiwan, ROC; 6 Department of Radiology, MacKay Memorial Hospital, MacKay Medical College, New Taipei City, Taiwan, ROC; 7 Faculty of Pharmacy, National Yang Ming Chiao Tung University, Taipei, 112304 Taiwan, ROC; 8 Cardiovascular Center and Division of Cardiology, Mackay Memorial Hospital, 92, Sec 2, Zhongshan North Road, Taipei, 10449 Taiwan, ROC

**Keywords:** Cardiology, Gastroenterology, Risk factors

## Abstract

Visceral adipose tissue (VAT) is associated with central obesity, insulin resistance and metabolic syndrome. However, the association of body-site specific adiposity and non-alcoholic fatty liver disease (NAFLD) has not been well characterized. We studies 704 consecutive subjects who underwent annual health survey in Taiwan. All subjects have been divided into three groups including normal (341), mild (227) and moderate and severe (136) NAFLD according to ultrasound finding. Pericardial (PCF) and thoracic peri-aortic adipose tissue (TAT) burden was assessed using a non-contrast 16-slice multi-detector computed tomography (MDCT) dataset with off-line measurement (Aquarius 3DWorkstation, TeraRecon, SanMateo, CA, USA). We explored the relationship between PCF/TAT, NAFLD and cardiometabolic risk profiles. Patients with moderate and mild NAFLD have greater volume of PCF (100.7 ± 26.3vs. 77.1 ± 21.3 vs. 61.7 ± 21.6 ml, P < 0.001) and TAT (11.2 ± 4.1 vs. 7.6 ± 2.6 vs. 5.5 ± 2.6 ml, P < 0.001) when compared to the normal groups. Both PCF and TAT remained independently associated with NAFLD after counting for age, sex, triglyceride, cholesterol and other cardiometabolic risk factors. In addition, both PCF and TAT provided incremental prediction value for NAFLD diagnosis. (AUROC: 0.85 and 0.87, 95%, confidence interval: 0.82–0.89 and 0.84–0.90). Both visceral adipose tissues strongly correlated with the severity of NAFLD. Compared to PCF, TAT is more tightly associated with NAFLD diagnosis in a large Asian population.

## Introduction

Non-alcoholic fatty liver disease (NAFLD) characterized by excessive fat infiltrations of liver becomes a major public health issue in the world due to increasing prevalence and the trend to cause pathological change including fibrosis and cirrhosis^1^. It is also associated with elevated triglycerides and reductions in the high density lipoprotein (HDL) cholesterol secondary to increases in the size of the very low density lipoprotein (VLDL), which are independent risk factors of metabolic syndrome and cardiovascular disease^2^.

Excessive visceral adipose tissue (VAT) is related to systemic inflammation, metabolic abnormalities including impaired glucose tolerance, hypertension, diabetes and heart failure^3^. However, regional-specific adiposity located over areas such as pericardial, peri-aortic root and thoracic peri-aortic were considered as neither sharing the same metabolic biochemistry nor contribute equally to central obesity^4^. Recently, a number of studies assessed the association between NAFLD and adipose tissue surrounding heart and thoracic descending aorta. Specifically, Iacobellis et al. suggested pericardial fat thickness had significant correlation with the severity of NAFLD in a study with 120 subjects of white and obese Caucasian^5^. Petta et al. reported that a higher pericardial fat thickness is associated with the severity of liver fibrosis in NAFLD subjects^6^. However, the relationship between the severity of NAFLD and 3D volume-measured regional-specific adipose tissue such as pericardial fat (PCF) and thoracic peri-aortic adipose tissue (TAT) in a large population remained largely unexplored. Therefore, our goal is to test the hypothesis that PCF and TAT, the CT-measured volume of adipose tissue, correlated with the severity of NAFLD in a large Asian population.

## Methods

### Study population

The study was approved by the Institutional Review Board of Mackay Memorial Hospital, Taipei, Taiwan. All participants signed written informed consent prior to examinations. Data were analyzed anonymously. From 2005 to 2012, a total of 719 consecutive subjects underwent cardiovascular health survey at our center that included a non-contrast enhanced computed tomography (CT) scan of the heart for coronary calcium scoring. A subset of 704 participants also had a liver ultrasound scan were eligible for the inclusion of the present study. Ultrasonography was performed using Philips EPIQ Ultrasound Machine. The images were interpreted by board certified gastroenterologists who were unaware of the clinical or laboratory data of the participants. Fatty liver was assessed, based on the presence of increased hepatic echogenicity making it distinguishable from the renal parenchyma of liver. Mild fatty liver was assessed as the minor increase in liver echogenicity. In moderate fatty liver, there were visual images associated with intrahepatic vessels, the slightly damaged diaphragm and the existence of increased liver organ echogenicity. Severe fatty liver was defined as the significant increase in hepatic echogenicity, poor penetration of posterior segment from the right lobe of the liver, poor or any visual images from the hepatic vessels and diaphragm. We defined NAFLD as fatty liver in individuals whose alcohol use disorders identification test (AUDIT) score was less than 8. Baseline demographics and medical history were obtained along with a detailed physical exam. Structured questionnaires were used to quantify self-reported alcohol consumption, smoking and physical activity. Subjects were excluded if they have any of the following issues: (1) heavy alcohol users, (2) who used statin during the previous year only, (3) presence of serological evidence of viral hepatitis or other chronic liver disease. Our definition for heavy alcohol user was according to the national institution of alcohol abuse and alcoholism, which is more than 4 drinks on any day or more than 14 drinks per week (for men) and consuming more than 3 drinks on any day or more than 7 drinks per week (for women). Among 704 study participants, 667 (94.7%) had NAFLD Fibrosis Score available, which may serve as a simple estimate on extent of fatty liver fibrosis^7^.

### Baseline anthropometrics and metabolic syndrome

A variety of anthropometric measures including height, weight, waist and hip circumferences were obtained. Resting blood pressures were measured by medical staff using a standardized sphygmomanometer. Anthropometric measures collected were height, weight, body mass index (BMI), waist and hip circumference. Standardized blood pressures were measured at rest by medical staff blinded to the other test results. Total body fat mass was measured by bioelectrical impedance using a Tanita-305 foot-to-foot body-fat analyzer (Tanita Corp., Tokyo, Japan). The definition of metabolic syndrome used a waist circumference cut-off of ≥ 90 cm and 80 cm for Taiwanese men and women, respectively. Additional criteria were: systolic blood pressure ≥ 130 mmHg or diastolic blood pressure ≥ 85 mmHg, triglyceride level ≥ 150 mg/dL, fasting blood sugar level ≥ 100 mg/dL, and HDL ≥ 40 and 50 mg/dL in men and women, respectively. The metabolic score therefore ranged from 0 to 5.

The presence of metabolic syndrome (MetS) was defined as a metabolic score of 3 or more. We also used Homeostatic Model Assessment for Insulin Resistance (HOMA-IR) as for the quantify method for evaluating insulin resistance.

### Pericardial (PCF) and thoracic periaortic adipose tissue volume (TAT)

Pericardial (PCF) and thoracic peri-aortic adipose tissue (TAT) volumes were quantified from the ECG-gated non-enhanced cardiac CT images using a dedicated workstation (Aquarius 3D Workstation, TeraRecon, San Mateo, CA, USA). The semi-automatic segmentation technique was developed for quantification of adipose tissue volumes. We traced the region of interest manually and defined adipose tissue as pixels within a window of − 195 HU to − 45 HU and a window centre of − 120 HU. PCF was defined as all adipose tissue located within the pericardial sac. TAT tissue was defined as all adipose tissue surrounding the thoracic aorta extending 67.5 mm caudally from the level of the bifurcation of pulmonary arteries. This approach has previously been validated^8,9^. The intra-observer and inter-observer coefficient of variation were 4.27%, 4.87% and 6.58%, 6.81% for PCF and TAT^9^.

### Statistical analysis

All the analyses were performed by using SPSS 15 (SPSS Inc., Chicago, IL). The characteristics of study subjects were expressed either as mean ± SD or frequency with percentage. Study subjects were divided into three groups according to their degree of fatty liver diagnosis: normal, mild, moderate and severe. Linear contrast in general linear model was used to examine the trend of each continuous variable across groups; Mantel–Haenszel Chi-squared test was used for categorical variables. Each P value for linear trend was reported.

Concerning with the ordinal nature of the fatty liver diagnosis, ordinal logistic regression was applied. The results of ordinal logistic regression are presented as the odds ratio (OR) and 95% confidence interval (CI) of being in a more severe fatty liver level for 1-unit change in serum parameters or for the presence or absence of medical history/life style variables.

The association of biomarkers—PCF and TAT—with fatty liver was assessed in different adjustment logistic models. In addition to these two biomarkers, models also included (1) age and gender; (2) age, gender, and established risk factors (3) age, gender, established risk factors, and life styles. Established risk factors were systolic blood pressure (SBP), fasting glucose, triglyceride, high-density cholesterol (HDL), cholesterol, eGFR, hypertension, diabetes, and hyperlipidemia. Life style factors contained regular exercise (yes vs. no), alcohol consumption (ever vs. never), and smoking status (ever vs. never). Each anthropometric factor—BMI, body fat, or waist circumstance—was further adjusted in Model 4, separately.

To identify the incremental values of PCF and TAT for the diagnosis of fatty liver beyond metabolic syndrome, likelihood ratio test was performed. Areas under ROC curve (AUC) and 95% CIs of each biomarker were reported to discriminate the prediction for fatty liver severity (moderate and severe vs. normal/mild) from metabolic syndrome.

### Ethics approval and consent to participate

All procedures were performed in accordance with the ethical standards of the institution and the 1964 Helsinki Declaration. The MacKay Memorial Hospital Group Ethics Committee approved our retrospective study (12MMHIS074).

## Results

### Characteristics of study subjects

There were 704 subjects enrolled from health examinations in this study. Majority of them were males (n = 527, 74.9%) and the mean age of them was 48.03 years old. The characteristics of the study subjects were summarized in Table 1. Most anthropometric measurements, serum parameters, medical history and life styles showed significant association with fatty liver diagnosis.

**Table 1:** Characteristics of study subjects by fatty liver diagnosis.

Characteristics	Fatty liver diagnosis			*P* for linear trend
	Normal (*n* = 341)	Mild (*n* = 227)	Moderate and severe (*n* = 136)	
**Anthropometric measure**				
Age (yr)	47.2 ± 7.9	47.2 ± 7.6	51.5 ± 9.5	< 0.001
Male gender, %	226 (66.3)	186 (81.9)	115 (84.6)	< 0.001
Body weight (kg)	61.5 ± 9.4	70.7 ± 9.0	77.3 ± 10.1	< 0.001
Height (cm)	165.3 ± 7.8	167.7 ± 7.1	167.2 ± 7.6	0.013
BMI (kg/m^2^)	22.4 ± 2.4	25.1 ± 2.4	27.6 ± 3.1	< 0.001
Percentage of body fat (%)	23.1 ± 5.5	26.4 ± 6.0	29.2 ± 7.1	< 0.001
Waist circumference (cm)	78.2 ± 8.0	85.0 ± 6.6	92.4 ± 7.2	< 0.001
Hip circumference (cm)	90.8 ± 5.3	93.9 ± 8.2	98.5 ± 6.3	< 0.001
Waist–hip ratio	0.86 ± 0.07	0.90 ± 0.05	0.94 ± 0.05	< 0.001
**Serum parameters**				
Systolic BP (mmHg)	115.7 ± 14.4	122.5 ± 16.1	129.8 ± 17.9	< 0.001
Diastolic BP (mmHg)	72.7 ± 10.2	77.5 ± 9.9	80.8 ± 10.7	< 0.001
Fasting glucose (mg/dL)	94.5 ± 17.5	99.8 ± 19.1	111.7 ± 34.3	< 0.001
Triglyceride (mg/dL)	109.2 ± 51.5	160.6 ± 84.4	178.2 ± 133.5	< 0.001
HDL-C (mg/dL)	56.6 ± 13.9	47.4 ± 10.8	46.2 ± 10.8	< 0.001
LDL-C (mg/dL)	122.3 ± 30.8	130.6 ± 29.4	130.0 ± 30.6	0.017
Cholesterol (mg/dL)	191.7 ± 34.2	196.6 ± 31.4	196.0 ± 34.9	0.205
AST/GOT (U/L)	21.5 ± 8.7	23.5 ± 8.4	30.5 ± 15.2	< 0.001
ALT/GPT (U/L)	23.2 ± 13.9	32.4 ± 17.2	45.5 ± 31.3	< 0.001
eGFR (mL/min/1.73 m^2^)	85.1 ± 16.0	84.2 ± 13.7	81.7 ± 18.5	0.039
Hs-CRP (mg/L)	0.15 ± 0.33	0.28 ± 0.60	0.34 ± 0.40	0.003
CRP (mg/dL)	0.36 ± 2.03	0.26 ± 0.27	0.37 ± 0.37	0.951
HOMA-IR Index	1.26 ± 0.97	1.63 ± 0.89	2.62 ± 1.95	< 0.001
NAFLD Fibrosis Score	− 2.72 ± 1.11	− 2.86 ± 1.08	− 2.07 ± 1.22	< 0.001
**Regional-specific visceral fat**				
PCF (mL)	61.7 ± 21.6	77.1 ± 21.3	100.7 ± 26.3	< 0.001
TAT (mL)	5.5 ± 2.6	7.6 ± 2.6	11.2 ± 4.1	< 0.001
**Underlying disease**				
Hypertension, %	45 (13.2)	60 (26.4)	60 (44.1)	< 0.001
Diabetes, %	67 (19.6)	54 (23.8)	54 (39.7)	< 0.001
Hyperlipidemia, %	12 (3.5)	18 (7.9)	10 (7.4)	0.041
Glucose-lowering drugs	65 (19.1)	53 (23.3)	54 (39.7)	< 0.001
**Life style**				
Exercise, %	33 (9.7)	19 (8.4)	12 (8.8)	0.688
Alcohol consumption, %	50 (14.7)	27 (11.9)	20 (14.7)	0.803
Smoking, %	56 (16.4)	48 (21.1)	37 (27.2)	0.007

Continuous variables were presented as mean and standard deviation; *BMI* body mass index, *BP* blood pressure, *HDL* high-density lipoprotein cholesterol, *LDL* low-density lipoprotein cholesterol, *eGFR* estimated glomerular filtration rate, *hs-CRP* high sensitivity C-reactive protein, *PCF* pericardial fat, *TAT* thoracic peri-aortic adipose tissue.

Elevated proportion of males was observed as fatty liver progressed (P < 0.001). The more severe diagnosis of fatty liver was, the greater values of anthropometric measurements were. These anthropometric measurements included age, body weight, BMI, percentage of body fat, waist circumference, hip circumference and waist–hip ratio (all P < 0.01). Similar trend was also found in the following serum parameters: systolic blood pressure, diastolic blood pressure, fasting glucose, triglyceride, AST/GOT, ALT/GPT, and hs-CRP (all P < 0.01). Subjects with more severe fatty liver tended to have higher values of PCF (61.7 vs. 77.1 vs. 100.7) or TAT (5.5 vs. 7.6 vs. 11.2), or higher proportion of hypertension/diabetes (all P < 0.001). As for life style variables, only smoking status revealed linear trend with fatty liver (P = 0.007). Conversely, more severe degree of fatty liver was associated with decreased level of high-density lipoprotein cholesterol (P < 0.001) and eGFR (P = 0.039). Though the P values of height, low-density lipoprotein cholesterol and hyperlipidemia among groups were significant, individual linear trend was not consistent across groups of fatty liver diagnosis (Table 1).

### Crude association of each variable with diagnosis of fatty liver

Table 2 presented the odds ratios (ORs) of each variable for fatty liver. More severe fatty liver was significantly associated with males and elders (both P < 0.001). For those who had greater values of BMI, percentage of body fat, waist circumference, hip circumference, SBP, DBP, fasting glucose, triglyceride, LDL-C, AST/GOT, ALT/GPT, Hs-CRP, or who had hypertension, diabetes, hyperlipidemia, smoking habit were significantly associated with more severe degree of fatty liver diagnosis (all P < 0.05). Increased PCF and TAT values were significantly associated with more severe fatty liver diagnosis with odds ratios of 1.05 and 1.51, respectively (both P < 0.001). In contrast, increasing HDL-C and eGFR levels were significantly associated with less severe fatty liver diagnosis (both P < 0.05). No significant association of cholesterol, CRP, exercise and alcohol consumption was found with fatty liver diagnosis.

**Table 2:** The association of clinical variables with fatty liver diagnosis among study subjects.

Characteristics	Crude OR	95% of CI	*P*
Age (yr)	1.04	1.02–1.06	< 0.001
Male gender	2.39	1.70–3.38	< 0.001
BMI (kg/m^2^)	1.69	1.58–1.81	< 0.001
Percentage of body fat (%)	1.13	1.10–1.15	< 0.001
Waist circumference (cm)	1.19	1.16–1.22	< 0.001
Hip circumference (cm)	1.16	1.13–1.20	< 0.001
Systolic BP (mmHg)	1.04	1.03–1.05	< 0.001
Diastolic BP (mmHg)	1.06	1.04–1.07	< 0.001
Fasting glucose (mg/dL)	1.03	1.02–1.04	< 0.001
Triglyceride (mg/dL)	1.009	1.006–1.011	< 0.001
HDL-C (mg/dL)	0.94	0.93–0.95	< 0.001
LDL-C (mg/dL)	1.01	1.00–1.01	0.002
Cholesterol (mg/dL)	1.004	0.999–1.008	0.090
AST/GOT (U/L)	1.07	1.05–1.09	< 0.001
ALT/GPT (U/L)	1.05	1.04–1.06	< 0.001
eGFR (mL/min/1.73m^2^)	0.990	0.981–0.999	0.047
Hs-CRP (mg/L)	2.06	1.22–3.49	0.007
CRP (mg/dL)	0.99	0.81–1.22	0.948
HOMA-IR Index	3.08	2.14–4.44	< 0.001
NAFLD Fibrosis Score	1.65	1.40–1.96	< 0.001
PCF (mL)	1.05	1.04–1.06	< 0.001
TAT (mL)	1.51	1.42–1.59	< 0.001
Hypertension	3.35	2.40–4.68	< 0.001
Diabetes	1.97	1.43–2.72	< 0.001
Hyperlipidemia	1.85	1.05–3.28	0.034
Exercise	0.89	0.55–1.46	0.655
Alcohol consumption	0.93	0.61–1.39	0.710
Smoking	1.60	1.13–2.25	0.008

*OR* adds ratio, *CI* confidence interval, *PCF* pericardial fat, *TAT* thoracic peri-aortic adipose tissue.

### The association of pericardial fat and peri-aortic fat with fatty liver in the various adjustment models

The effects of PCF and TAT based upon both univariate and multivariate models were shown in Table 3. An increase of SD in PCF or TAT was significantly associated with increased risk for being more severe fatty liver level (OR = 3.56, 4.42; P < 0.001) in the univariate model, respectively. Such association remained when age and gender were adjusted with odds ratio of 3.48 and 5.24, respectively (see model 1). Adjusting for both serum parameters and medical history, the significant associations of PCF and TAT with diagnosis of fatty liver were still observed but with slight smaller ORs of 3.02 and 3.58, respectively (see model 2). When further adjusting for lifestyle variables, the effect of PCF and TAT were not substantially impacted with odds ratios of 2.99 and 3.64, respectively (see model 3). In model 4, each additional anthropometric variable was introduced to assess the corresponding association of PCF and TAT with diagnosis of fatty liver. In other words, in model 4 despite the variables in model 3, we further adjusted for BMI, body fat, or waist circumstance, separately. Compared with the results of PCF in model 3, the ORs of PCF dropped to 1.86, 2.16 and 2.03 when BMI, body fat and waist circumference was adjusted in the Model, separately. Similar results were seen for TAT with ORs of 2.06, 2.48 and 2.35 in model 4. Though the ORs of PCF and TAT for fatty liver severity were decreasing as more variables were adjusted in the logistic models, the ORs were still statistically significant with P below 0.001 (Table 3).

**Table 3:** The association of pericardial fat (PCF), thoracic peri-aortic adipose tissue (TAT) with fatty liver diagnosis in various adjustment models.

Model	Pericardial fat (per SD)			Peri-aortic fat (per SD)		
	OR	95% of CI	*P*	OR	95% of CI	*P*
Unadjusted model	3.56	2.97–4.26	< 0.001	4.42	3.59–5.43	< 0.001
Model 1	3.48	2.87–4.22	< 0.001	5.24	4.09–6.71	< 0.001
Model 2	3.02	2.40–3.79	< 0.001	3.58	2.71–4.72	< 0.001
Model 3	2.99	2.38–3.75	< 0.001	3.64	2.75–4.82	< 0.001
Model 4 (BMI)	1.86	1.44–2.39	< 0.001	2.06	1.52–2.79	< 0.001
Model 4 (Body fat)	2.16	1.70–2.76	< 0.001	2.48	1.84–3.34	< 0.001
Model 4 (Waist circumstance)	2.03	1.59–2.61	< 0.001	2.35	1.74–3.17	< 0.001
Model 4 (HOMA-IR)	2.36	1.98–3.10	< 0.001	2.73	2.11–3.88	< 0.001

Model 1 adjusted for age, gender; Model 2: adjusted for age, gender, SBP, fasting glucose, triglyceride, HDL, cholesterol, eGFR, hypertension, diabetes, hyperlipidemia; Model 3: adjusted for age, gender, SBP, fasting glucose, triglyceride, HDL, cholesterol, eGFR, hypertension, diabetes, hyperlipidemia, life style (regular exercise, alcohol consumption, and smoking); Model 4: further adjusted for BMI, body fat, HOMA-IR or waist circumstance, separately.

*PCF* pericardial fat, *TAT* thoracic peri-aortic adipose tissue.

### Incremental value of pericardial fat and peri-aortic fat to the diagnosis of fatty liver beyond metabolic syndrome

**Figure 1:**
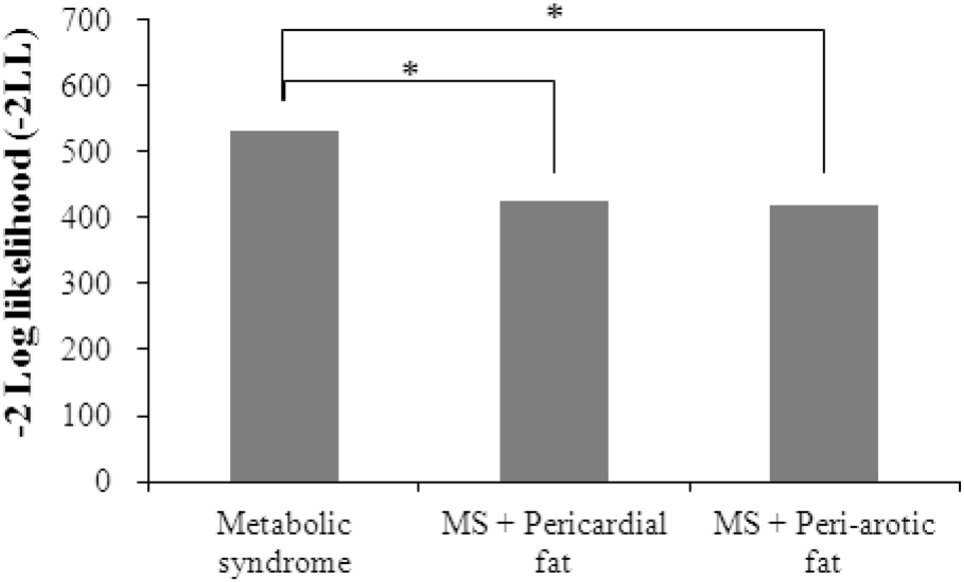
The incremental value of likelihood ratio test in discriminating fatty liver diagnosis (* indicates *P* values of delta LR test < 0.001).

**Figure 2:**
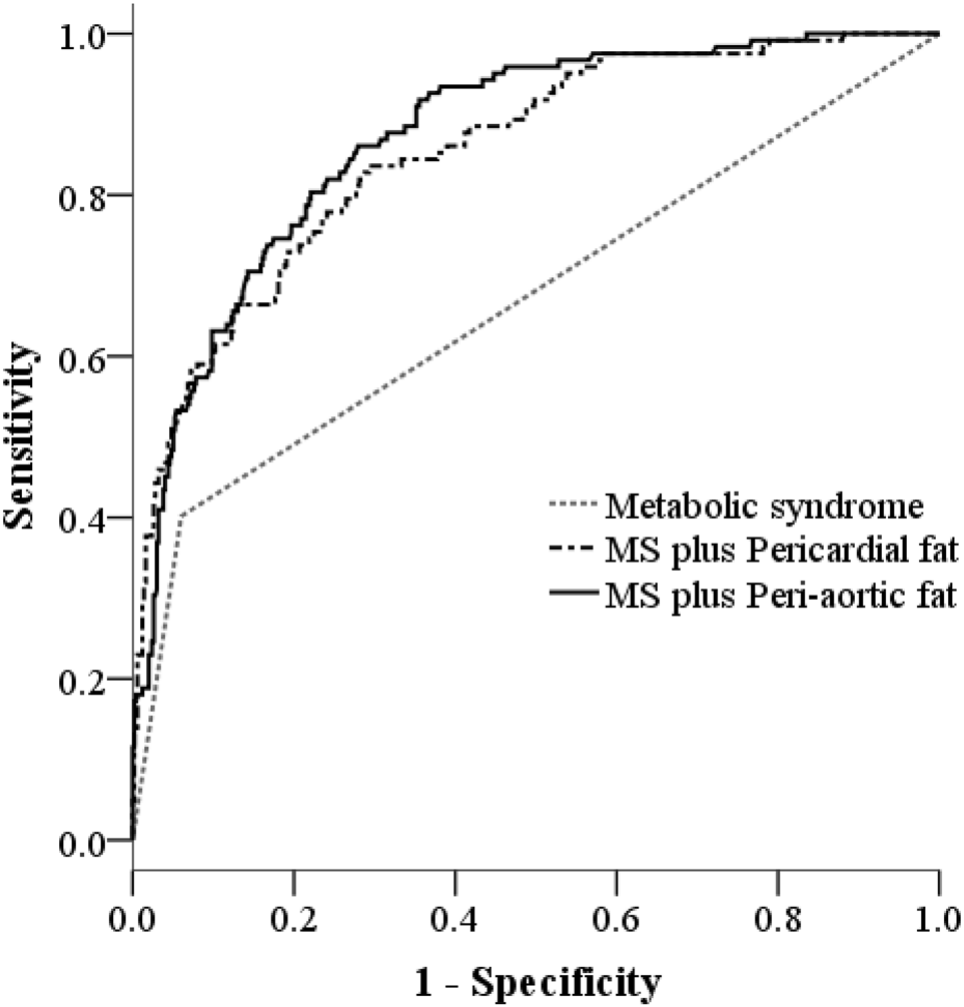
ROC curve analysis for metabolic syndrome, pericardial fat and thoracic peri-arotic fat in discriminating fatty liver diagnosis.

Table 4 listed the incremental values of PCF and TAT to the diagnosis fatty liver beyond metabolic syndrome. The AUC of Metabolic syndrome for fatty liver severity alone was 0.67 (95% CI = 0.61–0.73). When PCF was further included in the analysis, the AUC increased to 0.85 (95% CI = 0.82–0.89) with a significant P value based on LR test (△LR χ^2^ = 108.79, P < 0.001). Similarly, TAT along with metabolic syndrome showed increased AUC of 0.87 (95% CI = 0.84–0.90) with a significant LR test (△LR χ^2^ = 114.36, P < 0.001). The likelihood ratio tests were presented in Fig. 1. Figure 2 depicted the ROC curves of both biomarkers. It was clear that these biomarkers improved the prediction for fatty liver diagnosis as the ROC curves of metabolic syndrome with combination of PCF or TAT moved forward to the upper-left corner of the figure. To sum up, there was significant association of both PCF and TAT with diagnosis of fatty liver independent of metabolic syndrome.

**Table 4:** The incremental values of pericardial fat (PCF), thoracic peri-aortic adipose tissue (TAT) beyond metabolic syndrome in discriminating fatty liver diagnosis.

Predictor combination	AUC (c statistics)	95% CI of AUC	*P*	△LR χ^2^
Metabolic syndrome	0.67	0.61–0.73	< 0.001	–
Metabolic syndrome + PCF	0.85	0.82–0.89	< 0.001	108.79*
Metabolic syndrome + TAT	0.87	0.84–0.90	< 0.001	114.36*

*AUC* area under the ROC curve, *CI* confidence interval, *LR* likelihood ratio, which indicates reduction in deviance from the Metabolic syndrome only model; * indicates *P* values of delta LR test < 0.001.

*PCF* pericardial fat, *TAT* thoracic peri-aortic adipose tissue.

## Discussion

NAFLD is one of the most common diseases in the western world, affecting up to 15–20% of adult population. The definition of fatty liver is the hepatocyte contains more than 5% of triglycerides^10^. Several diagnostic tools are available for diagnosing fatty liver. Biopsy is the golden standard, but it is an invasive and may not universally be accepted by patients in clinical practice^11^. Nowadays, ultrasonography (US), due to its convenience and noninvasiveness, is most common tool for imaging diagnosis of fatty liver^12^.

Fatty liver is associated with obesity, insulin resistance and diabetes may cause chronic inflammation, adipose tissue remodeling, increased circulating level of pro-inflammatory cytokine (C-reactive protein, interleukin-6, monocyte chemotactic protein 1, and TNF-a)^1,13^, which is also metabolic syndrome pathogenesis. Although NHANES III cross-sectional data has shown that fatty liver is more likely to be a separate entity rather than an additional component of MS, fatty liver is more common in patients with obesity and MS^10^.

Although body mass index (BMI) is known as an independent predictor of NAFLD, visceral adipose tissue (VAT) which is associated with NAFLD even in non-obese subject is another important health issue related to obesity and metabolic syndrome^14^. On the other hand, in the past decade, the association of cardiovascular disease, metabolic syndrome and regional-specific VAT has been investigated. PCF located between the myocardium and visceral pericardium is an active endocrine organ with biochemical properties and reflects intra-abdominal fat^15^. TAT represents adipose tissue adjacent to descending thoracic aorta. Both PCF and TAT are visceral adipose tissue and may cause coronary calcification and atherosclerosis^16^. Several image studies including CT scanner^17^, Magnetic Resonance Image (MRI) and echocardiography are used to measure the thickness of pericardial, intra-thoracic or intra-abdominal adipose tissue^18,19^. Both epidemiological and physiological studies had demonstrated a strong association between excess adipose tissue and the presence of metabolic risk factors for coronary heart disease, including insulin resistance, impaired glucose tolerance, type 2 diabetes, dyslipidemia, and increased circulating inflammatory proteins^20,21^.

Independent positive association was observed between VAT and NAFLD^22^. And the reverser association between subcutaneous adipose tissue (SAT) and NAFLD has also been reported^23^. It is probably due to retaining adipose tissue in subcutaneous in human obesity could reduce overall and regional-specific VAT and improve insulin resistance^24^. A MRI study demonstrated the whole abdominal VAT volume and disproportion VAT/SAT in the lean subjects with NAFLD was as high and similar to VAT volume in both the overweight and obese subjects with or without NAFLD. However, the mechanism of VAT to fatty liver was uncertain. Some hypotheses were mentioned in previous studies^25^. It is believed that increased VAT directly involved in the pathogenesis of metabolic dysfunction because adipocytes in the visceral fat promote to release free fatty acids and the subsequent production of cytokines, such as adiponectin, interleukin-6, tumor necrosis factor-α, and leptin, and these adipocytokines flow directly into the liver because abdominal fat has a circulatory communication pathway to the liver via the portal vein. In addition, these adipocytokines may also induce systemic toxicity, insulin resistance and hepatic steatosis^20,23^.

In this study, we demonstrated that volume of regional-specific VAT including PCF and TAT had positive correlation with severity of fatty liver, anthropometric measures and serum parameter. TAT has stronger impact on fatty liver than PCF. It may be due to the different location of visceral fat. Accumulation of peri-vascular fat depots, such as TAT, may infiltrate to vascular by macrophages, inflammatory cytokines diffuse through arterial wall and directly released into the circulation with downstream effect^16^. The fat and cytokines would go through aorta to hepatic artery and directly induce fat accumulation, insulin resistance and cell remodeling, and eventually exacerbate fatty liver. As for PCF, which confined between the myocardium and visceral pericardium, would cause local inflammation and likely has direct effects on coronary atherosclerosis and cardiovascular disease, however, cause less effect on large vessel and its downstream effect^26^.

Recently, there are a few studies that focus on the same issue^27–29^. These studies were partially similar to our work. On one hand, they measured pericardial fat (PCF) and compared to NAFLD, CIMT and CAC comprehensively. However, our work provided additional valuable information about thoracic peri-aortic adipose tissue (TAT) and NAFLD. In fact, not only PCF and TAT, it seems that various regional-specific adipose tissue have different biological effects. In previous animal work, even perivascular fat surrounding thoracic and abdominal aorta have different effects on the physiology^30^. Therefore, in our humble opinion, our work provided the insight into the correlation between PCF, TAT and NAFLD. To our knowledge, this is the first study to evidence the positive correlation between volume of PCF and TAT and severity of NAFLD in Asian population. Compared to our results Iacobellis et al. reported PCF was significantly higher in obese subjects with NAFLD when compared to those without NAFLD^31^. But the case number was relatively small (164 including obese and nonobese) and PCF was measured in thickness instead of volume. In addition, Asian population had relative small body size than Caucasian in average may be more susceptible to ectopic fat related metabolic abnormality and easily resulted in obesity, insulin resistance and metabolic syndrome^32^. The previous study showed compared to non-Hispanic whites, the liver fat increase liver fat associated with reduced levels of accumulation of VAT and SAT in Japanese subjects, even in non-obese subjects^33^.

Compared to previous study by Petta, we measured the volume of pericardial fat by computed tomography rather than cardiac echography and we defined severity of fatty liver by echography rather than biopsy.

### Limitations

Several limitations must be considered when interpreting the results of the current study. First, our subjects were enrolled from health examination center. They were relatively young, male predominantly and healthy and the invasive procedure such as biopsy was less accepted. Therefore, the further analysis of the association of VAT and histologic findings of non-alcoholic steatohepatitis and hepatic fibrosis is not feasible. Second, this survey is retrospective and cross-sectional without clinical outcomes. Third, the method we used in the current study to measure the total body fat mass was bioelectrical impedance using a Tanita-305 foot-to-foot body-fat analyzer. However, there are more accurate machines such as an octopolar Bioelectrical Impedance, that can be consider in the future studies. Finally, there may be residual confounding from unmeasured factors. Future longitudinal cohort studies are needed to further validate our findings.

## Conclusion

The present study demonstrated regional-specific VAT is an independent measure to predict NAFLD, beyond the commonly used anthropometric parameters and serum markers, and have positive strong correlation with severity of NAFLD. In Asian populations, the association between TAT and NAFLD diagnosis is closer than that of PCF. These data add to our knowledge on possible pathophysiological mechanism involved in patients with NAFLD. Future studies are warranted to confirm these observations and to explore how these processes may be targeted to mitigate or prevent disease progression.

## Data Availability

The datasets generated during and/or analyzed during the current study are available from the corresponding author on reasonable request.
